# Presumed Caudal Cerebellar Artery Infarction in Three Cats: Neurological Signs, MRI Findings, and Outcome

**DOI:** 10.3389/fvets.2018.00155

**Published:** 2018-07-05

**Authors:** Arianna Negrin, Olivier N. J. Taeymans, Sarah E. Spencer, Giunio B. Cherubini

**Affiliations:** ^1^Dick White Referrals, Six Mile Bottom, United Kingdom; ^2^School of Veterinary Science, University of Bristol, Langford, United Kingdom

**Keywords:** feline, MRI, vascular, ischemic, cerebellum

## Abstract

Ischemic cerebrovascular disease (CVD) is a relatively common condition in dogs but infrequent in cats, with acute or peracute onset of non-progressive neurological signs. Cerebellar artery infarction appears to be very uncommon in cats, with only two cases reported affecting the rostral cerebellar artery (RCA). This study aims to report for the first time the neurological signs, magnetic resonance imaging (MRI) findings and outcome in three cats diagnosed with presumed caudal cerebellar artery (CCA) infarction. Unique presentation of vestibular signs associated with CCA in three cats and similarities between dogs and humans are discussed.

## Introduction

In cats, cerebellar infarcts have been rarely reported and only cases affecting the rostral cerebellar artery (RCA) territory (1) causing mainly typical cerebellar signs. Infarcts of the caudal cerebellar artery (CCA) have never been described in cats. In dogs CCA infarcts are uncommonly reported and associated with vestibular signs (2). Differently, within the cerebrovascular accidents affecting human beings, cerebellar infarct involving the posterior-inferior cerebellar artery (PICA) are not uncommon (3). This artery supplies the posterior-inferior part of the cerebellar hemispheres and the flocculonodular lobe (3). Common clinical signs of PICA infarcts are mainly vestibular (balance loss, vertigo, and nystagmus) (3).

The present study describes for the first time the clinical findings, magnetic resonance imaging (MRI) characteristics and follow up of presumptive CCA infarct in three cats, discussing potential similarities with canine CCA and human PICA infarctions.

A standardized MRI protocol was used in all cats to assess the brain including the following sequences: fast-spin T2, T2 fluid attenuated inversion recovery (FLAIR), T2^*^ gradient-echo, spin echo T1, spin echo T1 post-contrast in transverse planes and fast-spin echo T2 in sagittal plane.

A written informed consent was obtained from the owners of each patient for publication.

### Case 1

An 11-year-old male neutered domestic shorthair cat was presented for investigation of acute vestibular signs. The owner reported a left sided head tilt and loss of balance observed a few hours prior to presentation. Physical examination was unremarkable while a left-sided head tilt, broad-based pelvic limb stance, and a mild intermittent intention head tremor was observed on neurological examination. Vestibular ataxia with falling to the left, decreased postural reactions in all four limbs (paw positioning, hopping, and extensor postural trust) and bilateral positional vertical nystagmus were also recorded. A left central vestibular syndrome was suspected.

Hematology, biochemistry, fasting bile acids, thyroxine levels, and urinalysis were unremarkable. Feline leukemia virus antigen and feline immunodeficiency virus antibody tests were negative (SNAP FIV/FeLV Combo test; IDEXX, Maine). Systolic blood pressure measured upon admission using Doppler sphygmomanometry was considered within normal limits for a hospital environment (mean reading = 151 mmHg).

**Figure 1:**
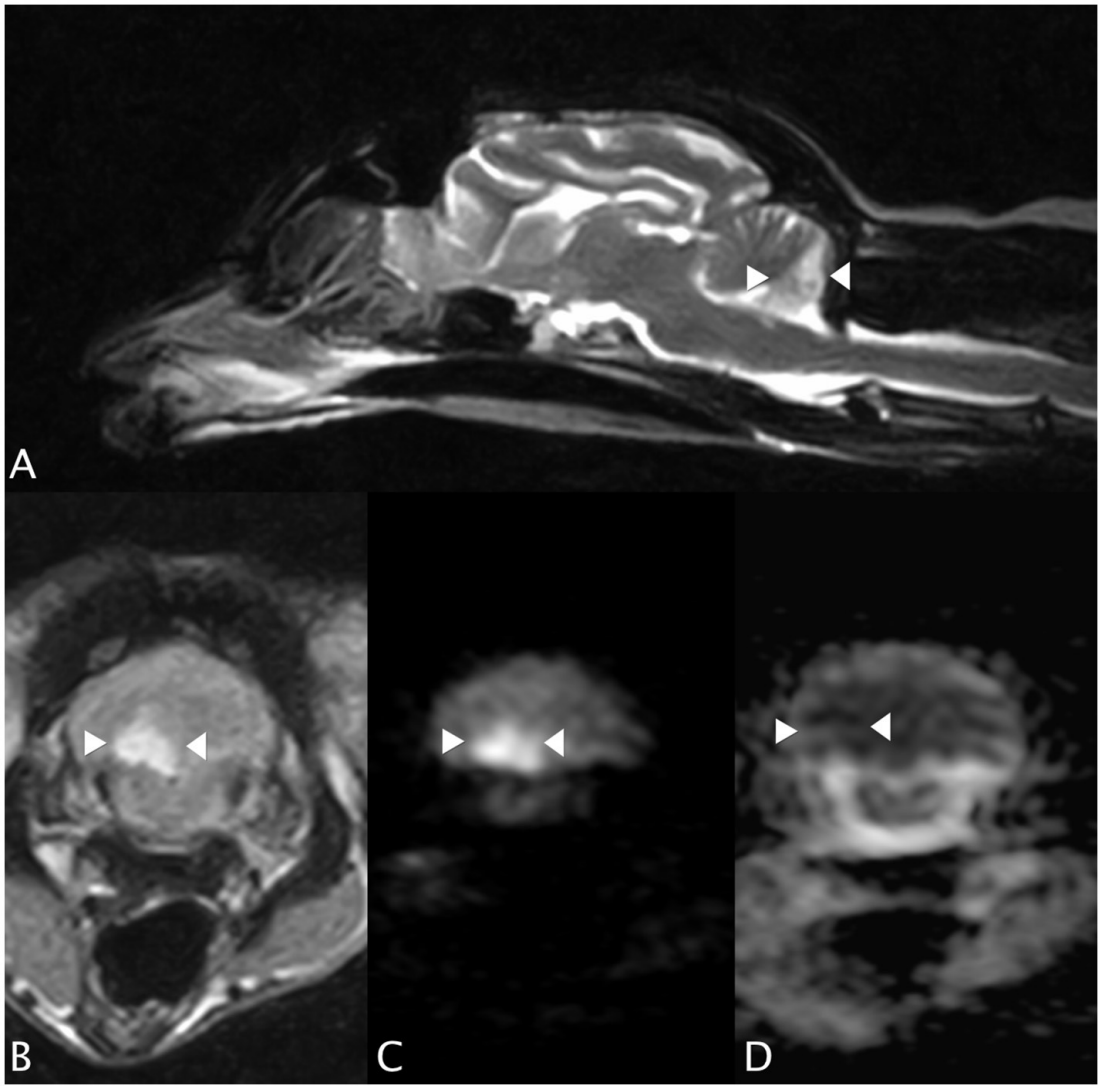
Case 1. **(A)** Sagittal T2-weighted MRI image, showing a sharply-demarcated hyperintense lesion in the caudal cerebellar hemisphere and vermis. There is no apparent mass effect. **(B)** Transverse T2 FLAIR MRI image (at the level of the lesion seen in **(A)**, patient's left sided is on the right hand side of the picture). The lesion is wedge-shaped, hyperintense and confined to the right cerebellum. **(C)** Transverse diffusion-weighted image (DWI) and **(D)** corresponding apparent diffusion coefficient (ADC) MRI images. Patient's left sided on R hand side of the picture. The arrowheads highlight the lesion, which has restricted diffusion (hyperintensity) on the DWI and a reduced ADC (hypointensity), typical of an acute infarct.

An MRI study of the brain (0.4T Hitachi Aperto Lucent MR scanner, Tokyo) was performed 24 h after the onset of neurological signs. A single, sharply-demarcated focal cerebellar lesion was identified in the caudal third of the right cerebellar hemisphere and lateral medulla oblongata (Figure 1A). The lesion was isotense on T1-weighted images, and hyperintense on T2-weighted images (Figures 1A,B) and T2 FLAIR images. Diffusion-weighted images (DWI) showed a well-demarcated area of uniform hyperintensity with a corresponding area of reduced diffusion (hypointensity) on an apparent diffusion coefficient (ADC) map (Figures 1C,D). No mass effect was seen. There was minimal enhancement with gadobutrol (Gadovist, Bayer, UK) administration (0.1 mmol/kg, IV). Based on the MRI appearance and presence of restricted diffusion, history, and neurological localization, a presumptive diagnosis of a non-hemorrhagic right CCA infarction, resulting in paradoxical central cerebellar syndrome, was made.

The cat was hospitalized for monitoring and supportive care. Oral antioxidant/multivitamin therapy (Aktivait, Vetplus, UK) was initiated as supportive trial therapy. The cat's neurological status progressively improved over 4 days and he was discharged with mild head tilt. No neurological signs were present at 1 month re-examination and antioxidant/multivitamin therapy was discontinued.

### Case 2

A 4-year-old female spayed domestic short hair cat was referred with a 24 h history of acute vestibular signs. Physical examination was unremarkable. Neurological examination revealed head tremors, left-sided head tilt and generalized vestibular ataxia with a tendency to fall to the left. Mild postural reactions deficits (in hopping and extensor postural trust tests) in the left pelvic limb and positional horizontal nystagmus on left eye were observed. Neuroanatomical localization suggested a left-sided central vestibular lesion, most likely central-cerebellar localization.

Hematology and serum biochemistry revealed hypercholesterolemia (5.6 mmol/l; RI 1.9–3.9) and a marked increased creatinine kinase (2405 IU/L; RI 0–152). Thyroid testing was consistent with non-thyroidal illness (thyroxine < 12.9 nmol/l [RI 15–50]; thyroid-stimulating hormone (TSH) 0.07 ng/ml [RI 0.0–0.32]). *Toxoplasma gondii* serology in blood (IgM and IgG) was negative.

**Figure 2:**
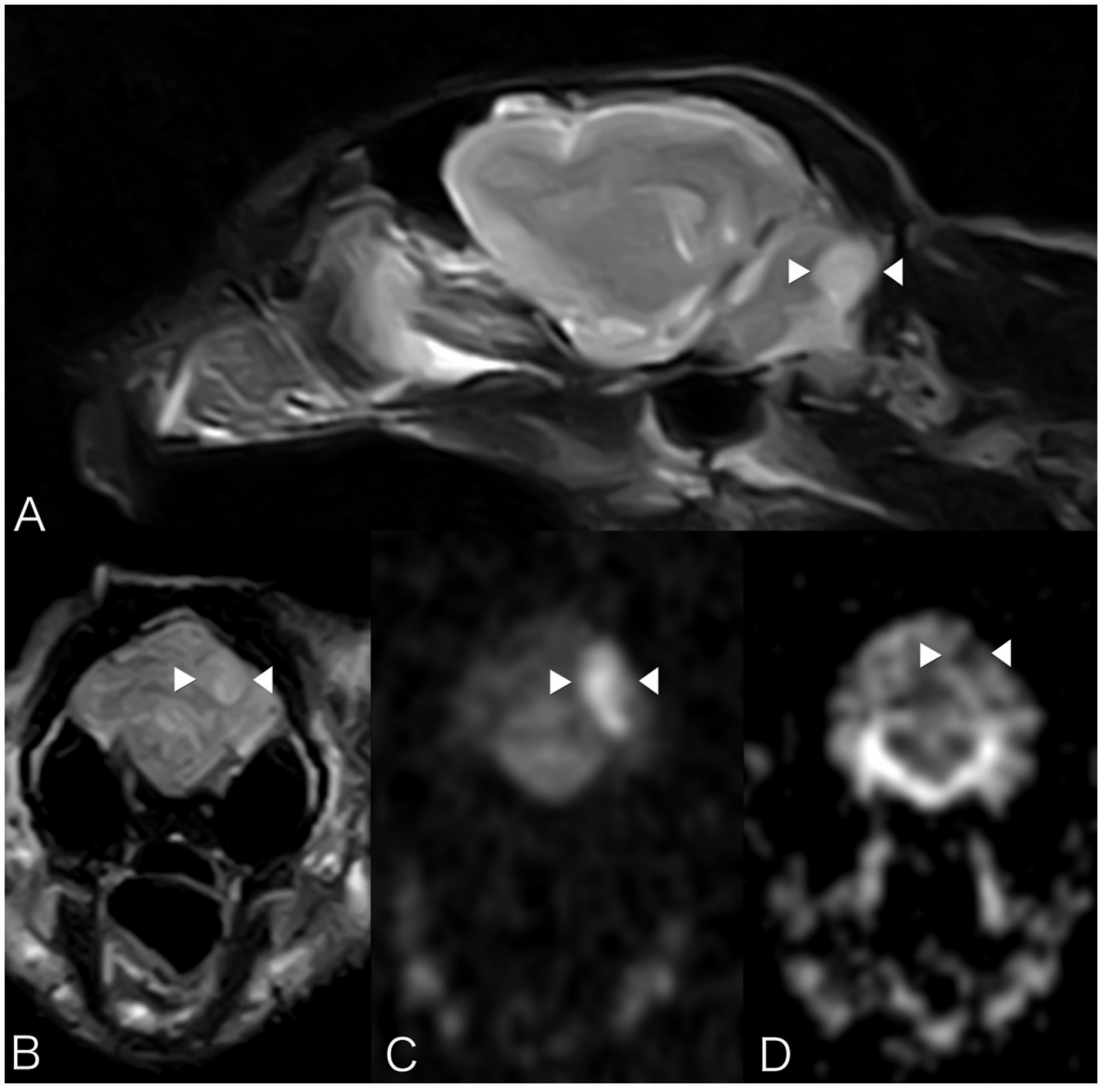
Case 2. MRI images show a focal well-defined hyperintense lesion on T2-weighted **(A)** and T2 FLAIR sequences **(B)** caudally in the left cerebellar hemisphere. Molecular diffusion is restricted on DWI images **(C)** and corresponding ADC map **(D)**. Patient's left sided is on the right hand side of the picture. Arrowheads underline the lesion described.

Brain MRI showed a focal well-defined hyperintense lesion on T2-weighted (Figures 2A,B) and T2 FLAIR sequences in the caudal left cerebellar hemisphere. Diffusion was restricted on diffusion-weighted sequences and corresponding ADC map (Figures 2C,D). A thin rim of contrast enhancement was seen around the margin of a cerebellar folium. No mass effect was observed. Findings were compatible with a non-hemorrhagic left CCA infarct. Spinal MRI (performed due to the left pelvic limb proprioceptive deficits) revealed facet hypertrophy causing dorsal impingement at T13-L1. Cisternal cerebrospinal fluid (CSF) analysis revealed mild neutrophilic pleocytosis (nucleated cell count 12cells/ul [RI 0–5]; total proteins were normal). Echocardiography, thoracic radiography, and abdominal ultrasound were unremarkable. The cat was discharged 48 h subsequently with mild generalized vestibular ataxia only. On telephonic follow-up 3 months later the cat's vestibular signs had completely resolved.

### Case 3

An 8-year-old male neutered Persian cat was presented for neurological evaluation of acute onset vestibular ataxia and abnormal mentation. Physical examination revealed a grade II/VI systolic heart murmur but was otherwise unremarkable. Neurological examination revealed generalized vestibular ataxia with marked loss of balance, generalized postural reactions deficits (hopping and paw positioning tests), absent oculocephalic reflex, and ventrolateral positional strabismus in both eyes. The neuroanatomical localization was compatible with a central vestibular lesion.

**Figure 3:**
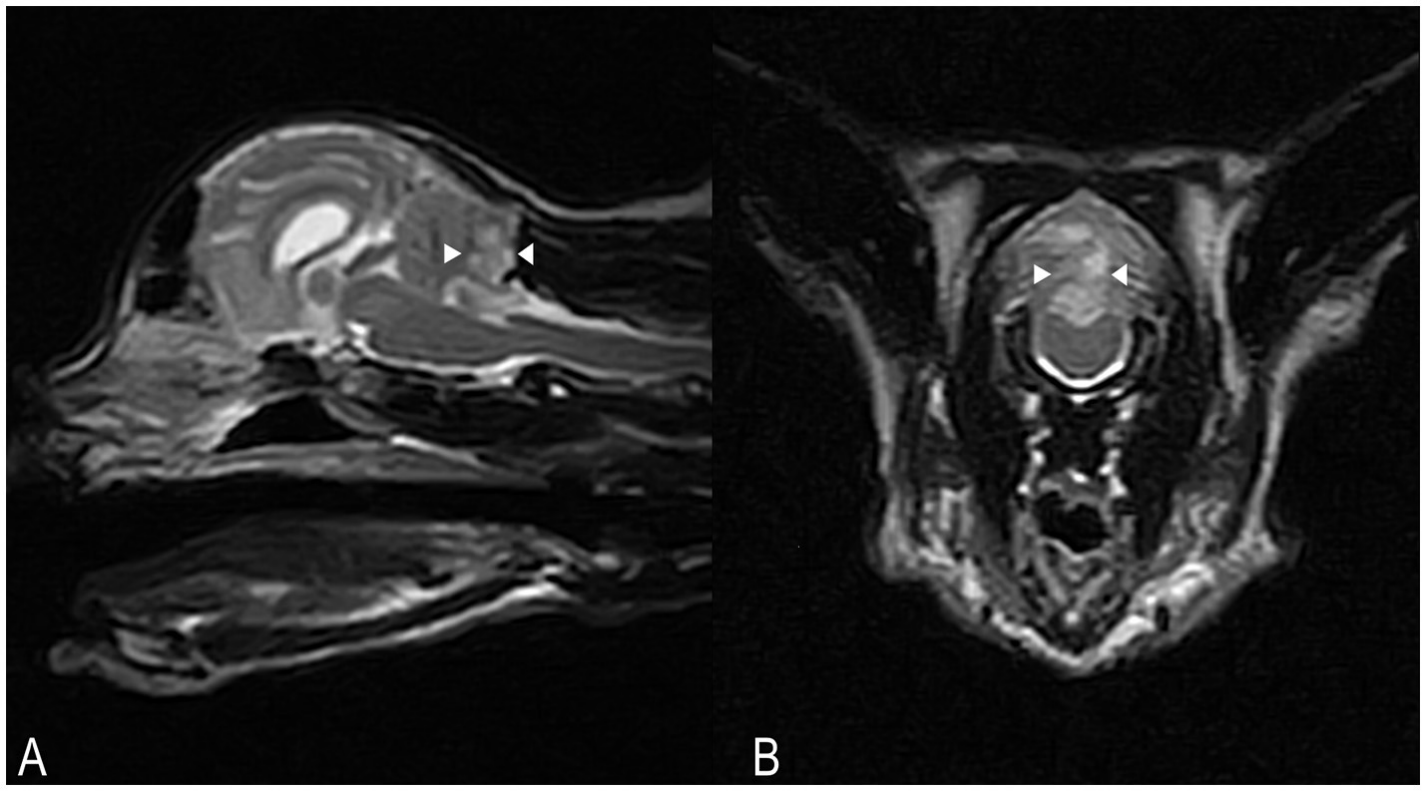
Case 3. SAG FSE T2 **(A)** and TRANS FSE T2 **(B)** MRI images. A poorly defined area of homogeneously increased T2 signal intensity (arrowheads) compared to normal cerebellar gray matter, measuring approximately 8 × 13 × 6 mm in size, is affecting the caudal aspects of the cerebellar vermis and right cerebellar hemisphere. In **(B)**, patient's left sided is on the right hand side of the picture.

Hematology and serum biochemistry were unremarkable. On MRI, a poorly defined area (8 × 13 × 6 mm) of homogeneously increased T2 and T2 FLAIR signal hyperintensity compared to normal cerebellar gray matter, affecting the caudal aspect of the cerebellar vermis and right cerebellar hemisphere was observed (Figures 3A,B). This lesion had a low signal in T1-weighted images, and there was no abnormal contrast enhancement. There was no signal void observed on T2^*^ weighted images. An acute non-hemorrhagic territorial cerebellar infarct affecting the perfusion area of the right CCA was suspected. Cisternal CSF analysis revealed a normal cell count and population but increased total protein at 0.68 g/l (RI < 0.25). RT-PCR for infectious diseases (*Toxoplasma gondii*, feline coronavirus and feline herpesvirus) in CSF was negative. Oral antioxidant/multivitamin therapy (Aktivait, Vetplus, UK) was instituted for 1 month. The cat's neurological status progressively improved over 4 days and he was discharged with markedly improved generalized ataxia and a mild head tilt. At 1 month follow-up no neurological signs were observed and therapy was discontinued.

## Discussion

Cerebrovascular brain diseases are rarely reported in cats (1, 4) and in relation to the cerebellum, only RCA infarction has been reported to date (1). To the authors' knowledge this study reports the first three cases of feline CCA infarction. In dogs, CCA infarction has only been reported in two dogs (2, 5), and therefore appears to be less common than RCA infarction (6, 7). Humans with PICA infarction, deemed the equivalent of the CCA (2), have a poorer prognosis than infarcts involving the anterior cerebellar circulation, due to higher tendency of severe mass effect and brainstem compression (3). The vascular supply of the canine and feline brain differs and this may contribute to differences in cerebrovascular disease incidence and distribution between species (2). Both the feline and canine cerebellum are supplied by two pairs of arteries: the RCA, which supplies the rostral part of the cerebellar hemispheres, vermis, and dorsolateral brainstem, and the CCA, which vascularizes the caudoventral cerebellum and lateral medulla oblongata (8–10). The CCA is a branch of the basilar artery in the dog, while in the cat it is supplied by the maxillary artery via arterial circle and a rete mirabile (8, 9).

MRI findings in this case series corresponded to neurological localization and were typical of previously reported acute non-hemorrhagic infarcts, with focal wedge-shaped T1-weighted isointense, T2-weighted and FLAIR hyperintense lesion (11). MRI techniques provide additional diagnostic support in distinguishing between acute and historic lesions and from peripheral to central vestibular localization. Diffusion weighted imaging (DWI) and ADC sequences increase the sensitivity and specificity in differentiating an acute from a chronic cerebrovascular disease. The ischemic core of an infarct appears hyperintense on DWI and can be differentiated from other T2-hyperintense lesions due to hypointensity on ADC map, which represents cytotoxic oedema (7).

No specific therapy is known to affect the outcome of idiopathic cerebrovascular diseases; instead treatment is supportive and aims to control any underlying disease. Although there is no evidence for antioxidant therapy use in cerebrovascular disease, increased production of reactive oxygen species is known to occur subsequent to infarction due to ischemia and necrosis (12). For this reason a trial therapy with antioxydants/multivitaminc complex has been used in these cases, however real efficacy is still unclear in human and veterinary medicine.

The reported cases all survived the acute cerebrovascular incident in in agreement with previous reports of cryptogenic non-hemorrhagic ischemic cerebrovascular disease in dogs, where no anti-oxidants were supplemented. (2, 5, 6). It differs, however, from the poor prognosis previously reported in RCA infarction in two cats, possibly due to the presence of underlying disease in these cases (1). Cerebrovascular disease was deemed cryptogenic in 12.5% of cats in the largest feline study (4), however presence of a concurrent disease is associated with a poorer prognosis in veterinary medicine and increased likelihood of subsequent recurrent infarction (6, 7). Feline ischemic cerebrovascular disease is commonly associated with an underlying disease, including hypertension, hyperthyroidism, neoplasia, cardiac disease, and chronic nephropathies (1, 4). In both reported cases of feline RCA infarction, concurrent predisposing disease (pulmonary adenocarcinoma and cardiac disease) was present (1). Complete screening for concurrent disease in the present cases was declined in two cases. An underlying pathology including occult cardiomyopathy, could therefore not be ruled out, however no abnormalities were detected on cardiac auscultation in these two cases and from the history all cats were reported healthy prior to presentation.

## Concluding remarks

This study supports accounts of cerebrovascular disease as a differential diagnosis of central vestibular signs and describes, for the first time, the clinical signs of cats with CCA infarction. Moreover, the diagnostic value of MRI, including DWI and ADC sequences, is highlighted. Despite differences in cerebral vascularization, CCA infarction can occur in cats as well as dogs and it appears to be associated with a good prognosis.

## Author contributions

All authors listed have made a substantial, direct and intellectual contribution to the work, and approved it for publication.

### Conflict of interest statement

The authors declare that the research was conducted in the absence of any commercial or financial relationships that could be construed as a potential conflict of interest.
